# Comparative analysis of gut microbiota associated with body mass index in a large Korean cohort

**DOI:** 10.1186/s12866-017-1052-0

**Published:** 2017-07-04

**Authors:** Yeojun Yun, Han-Na Kim, Song E. Kim, Seong Gu Heo, Yoosoo Chang, Seungho Ryu, Hocheol Shin, Hyung-Lae Kim

**Affiliations:** 1 Department of Biochemistry, Ewha Medical Research Institute, School of Medicine, Ewha Womans University, 1071, Anyangcheon-ro, Yangcheon-gu, Seoul, 07985 South Korea; 2 Wide River Institute of Immunology, Seoul National University, Seoul, South Korea; 3 Center for Cohort Studies, Total Healthcare Center, Kangbuk Samsung Hospital, School of Medicine, Sungkyunkwan University, Seoul, South Korea

**Keywords:** Gut microbiota, Body mass index (BMI), Obesity

## Abstract

**Background:**

Gut microbiota plays an important role in the harvesting, storage, and expenditure of energy obtained from one’s diet. Our cross-sectional study aimed to identify differences in gut microbiota according to body mass index (BMI) in a Korean population. 16S rRNA gene sequence data from 1463 subjects were categorized by BMI into normal, overweight, and obese groups. Fecal microbiotas were compared to determine differences in diversity and functional inference analysis related with BMI. The correlation between genus-level microbiota and BMI was tested using zero-inflated Gaussian mixture models, with or without covariate adjustment of nutrient intake.

**Results:**

We confirmed differences between 16Sr RNA gene sequencing data of each BMI group, with decreasing diversity in the obese compared with the normal group. According to analysis of inferred metagenomic functional content using PICRUSt algorithm, a highly significant discrepancy in metabolism and immune functions (*P* < 0.0001) was predicted in the obese group. Differential taxonomic components in each BMI group were greatly affected by nutrient adjustment, whereas signature bacteria were not influenced by nutrients in the obese compared with the overweight group.

**Conclusions:**

We found highly significant statistical differences between normal, overweight and obese groups using a large sample size with or without diet confounding factors. Our informative dataset sheds light on the epidemiological study on population microbiome.

**Electronic supplementary material:**

The online version of this article (doi:10.1186/s12866-017-1052-0) contains supplementary material, which is available to authorized users.

## Background

The growing incidence of obesity and obesity-associated complications, including diabetes, cardiovascular disease, and stroke, is a major public health concern worldwide [1]. The etiology of obesity, which implies an energy imbalance between calories consumed and expended, is complicated by biological and environmental factors [2, 3]. Recently, a large number of studies have demonstrated that gastrointestinal bacteria can interplay with diet in the development and propagation of obesity [4].

There have been considerable advances in determining possible mechanisms underlying gut microbiota-induced obesity [5–7]. These mechanisms contain a key feature of increased energy production/absorption; for example, short-chain fatty acid (SCFA)-producing bacteria can ferment indigestible dietary fiber and hydrogentrophs with the importance of H2 removal, an end product of bacterial fermentation [8–11]. Moreover, changes in metabolic pathways caused by intestinal dysbiosis, such as de novo lipogenesis in liver [12–14], can induce increased adiposity by host gene suppression [6]. In addition, the induction of low-grade inflammation by increased endotoxin exposure through gut leakage [15] and the effects of appetite and satiety regulation by leptin signaling on gut-brain axis [13] have been proposed as candidate pathways leading to obesogenic environments [15, 16].

However, the results of many articles speculating on the potential associations between gut microbiota and obesity are conflicting and have not been replicated in clinical studies. These shortcomings prevent designation of a consistent pattern of human gut microbiota that correlates with obesity [4, 17, 18]. Therefore, the challenge to incorporate assessment of microbiomes into epidemiologic studies remains and is critical. Surprisingly, there is a lack of statistically significant study with a large sample size in gut microbiota studies. The large sample sizes in epidemiologic studies will provide increased statistical power and help to reveal significant findings involved with human-associated microbiota [19].

Here we examined the correlation between the gut microbiota and body mass index (BMI) in relatively large sample size of Asian population. This study could contribute to further population-based association study using microbiota data.

## Methods

### Study subjects

**Fig. 1:**
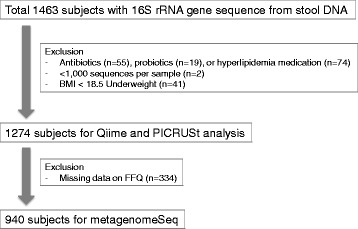
Flow chart of study subjects

The study used data from a total of 1463 subjects who were enrolled in the Kangbuk Samsung Health Study, which is a cohort study of Korean men and women who underwent a comprehensive annual or biennial examination at Kangbuk Samsung Hospital Total Healthcare Screening Centers in Seoul, South Korea, between June and September 2014 [20, 21]. The datasets provided the age, weight, and height for BMI (kg/m^2^) determination as well as dietary status (Table 1). The supplemental information regarding metabolic status of study groups is also shown in Table 1. We didn’t exclude total 42 type 2 diabetes (T2DM) patients including patients under medication, because of no significant difference between the BMI groups (Table 1). We excluded 141 participants because they had used: antibiotics within 6 weeks prior to enrollment (*N* = 55), cholesterol-lowering medications (*N* = 74), or probiotics (*N* = 19) within 4 weeks prior to enrollment (Fig. 1). The BMI was classified into categories of underweight (BMI < 18.5), normal (18.5 ≤ BMI < 23), overweight (23 ≤ BMI < 25), and obese (BMI ≥ 25) according to the revised Asia-Pacific BMI criteria by the World Health Organization Western Pacific Region [22]. Underweight subjects were excluded from this study (*n* = 41, Fig. 1). Dietary consumption was assessed using a 103-item self-administered food frequency questionnaire (FFQ) designed for use in Korea [23]. Dietary intake data were collected at the same day of the health checkup using the validated FFQ, which was designed to measure a participant’s usual consumption of foods and food groups during the previous year. This nutrient intake data has been validated in previous publications [24]. The variables selected for this study were total energy, carbohydrate, fiber, fat, and protein. Only subjects within three standard deviations of the mean value of the log-transformed energy intake were included when nutrients adjustments were needed (the missing data of nutrients was 334; Fig. 1). Nutrients variables were applied as residuals from the regression model, with absolute nutrient intakes as the dependent variables and total energy intake as the independent variable [25].

**Table 1:** Characteristics of the study population categorized by BMI

	Normal	Overweight	Obese	Trend *P* value^a^	Total
Subjects^b^	529	326	419		1274
Male%	41.6	72.4	85.0		63.7
Age (years)	45.3 (9.3)	46.3 (9.0)	45.8 (8.5)	0.302	45.7 (9.0)
BMI (kg/m^2^)	21.1 (1.4)	24.0 (0.6)	27.2 (2.1)	<0.0001	23.8 (3.0)
Fat mass (kg)	13.9 (3.0)	16 .9 (3.7)	22.0 (5.0)	<0.0001	17.5 (5.3)
Glucose (mg/dl)	92.6 (11.1)	95.9 (14.8)	98.8 (17.3)	<0.0001	96.2 (15.3)
Triglycerides (mg/dl)	94.5 (49.6)	119.7 (70.1)	147.5 (84.3)	<0.0001	119.9 (72.1)
HDL cholesterol (mg/dl)	62.6 (14.5)	55.4 (13.6)	49.8 (11.2)	<0.0001	56.3 (14.2)
Systolic BP (mmHg)	104.1 (11.7)	110.8 (11.7)	116.8 (13.0)	<0.0001	110.2 (13.1)
Diastolic BP (mmHg)	67.8 (9.1)	71.3 (9.2)	75.6 (10.2)	<0.0001	71.3 (10.0)
Insulin (ulU/ml)	4.2 (2.4)	5.4 (3.0)	7.5 (4.7)	<0.0001	5.7 (3.8)
HbA1c (%)	5.5 (0.4)	5.6 (0.4)	5.6 (0.5)	0.180	5.6 (0.5)
HOMA-IR	1.0 (0.6)	1.3 (0.9)	1.9 (1.4)	0.003	1.4 (1.1)
Hypertension^b^	14	19	56	<0.0001	89
Type 2 Diabetes (T2DM)^b^	12	15	15	0.191	42
Med. Of T2DM^b^	10	7	9	0.965	26
Subjects with nutrient information^b^	387	245	308		940
Male%	40.8	71.8	84.7		65.4
Total calorie (kcal/day)	1423.5 (603.8)	1543.7 (679.8)	1596.9 (669.5)	0.001	1512.3 (649.9)
Carbohydrate (g/day)^c^	251.2 (47.8)	257.3 (46.7)	251.2 (50.2)	1.000	253.0 (46.7)
Fiber (g/day)^c^	13.1 (5.6)	12.8 (5.5)	12.4 (5.0)	0.064	12.8 (5.4)
Fat (g/day)^c^	27.0 (11.9)	26.8 (12.7)	28.3 (12.6)	0.211	27.4 (12.2)
Protein (g/day)^c^	49.7 (11.0)	50.0 (10.6)	51.0 (10.7)	0.156	50.3 (10.7)

Data are presented as mean (SD)

*BMI* body mass index, *HDL* high-density lipoprotein, *BP* blood pressure, *HbA1c* Hemoglobin A1c, *HOMA-IR* homeostasis model assessment-estimated insulin resistance (insulin (μU/mL) × glucose (mg/dL)/405)

^a^Trend *P* value from multiple logistic regression analysis

^b^Count data

^c^Nutrients adjusted for energy using the residual method

### DNA extraction and sequence data generation

The 16S rRNA genes were extracted and amplified from stool specimens using the MO-BIO PowerSoil DNA Isolation Kit (MO-BIO Laboratories) according to the manufacturer’s instructions. Amplification and sequencing were performed as described previously for analysis of bacterial communities [26]. Briefly, the V3-V4 domain of bacterial 16S rRNA genes was amplified using primers F319 (5′- TCGTCGGCAGCGTCAGATGTGTATAAGAGACAG) and R806 (5′–GGACTACHVGGGTWTCTAAT–3′). Each primer was modified with Nextera® XT kit (Illumina, Inc.) to contain a unique 8-nt barcode index by combination (16 of S and 24 of N). Polymerase chain reactions (PCRs) comprised a 5 ng/μL DNA template, 2 × KAPA HiFi HotStart Ready Mix (KAPA Biosystems), and 2 pmol of each primer. Reaction conditions were an initial 95 °C for 3 min, followed by 25 cycles of 95 °C for 30 s, 55 °C for 30 s, and 72 °C for 30 s, and a final extension of 72 °C for 5 min. After PCR cleanup and indexing, sequencing was performed on the Illumina MiSeq platform (Illumina, Inc.) according to the manufacturer’s specifications. The 100 bp of overlapping paired-end reads were merged using pandaseq (version 2.7). Only data from Illumina reads with a length of >300 bp were retained for further analysis. Chimeric sequences were filtered out by UCHIME algorithm in USEARCH platform which performs both de novo chimera and reference based detection (USEARCH v6.1.544).

### Sequence analysis

Microbial operational taxonomic units (OTUs) and their taxonomic assignments were obtained using default settings in the QIIME version 1.9 and by closed reference mapping at 97% similarity against representative sequences of Greengenes (version 13_8). We used all default settings in QIIME 1.9 for OTU mapping and the pre-assigned taxonomy for the Greengenes OTU representative sequences. As recommended for Illumina-generated data [27], we removed OTUs comprised <0.005% of reads in the total data set. Samples with <1000 sequences per sample (*n* = 2) were considered failures and filtered out (Fig. 1). Finally, total 1274 subjects with a mean of 26,024 (+/−18,528) sequences per sample were included for the QIIME analysis. Alpha and beta diversity on Cumulative Sum Scaling (CSS) normalized OTU tables to assess phylogenetic diversity (PD) metrics were calculated by QIIME [28]. The PD metrics provide a measure of alpha diversity of taxa present based on phylogenetic tree within subjects, while the weighted UniFrac distance metrics reflects the similarity between bacterial communities between subjects, so called beta diversity. The significant difference between categories in alpha diversity (PD) and beta diversity (weighted UniFrac) was compared by creating boxplots with a two-sided Student’s two-sample t-test. The analysis of similarities (ANOSIM) on beta diversity was applied to test the difference of distance metrics by grouping, and a *P* value was calculated by 999 Monte Carlo permutation non-parametric tests.

### PICRUSt analysis

The PICRUSt approach was used to evaluate the functional potential of microbial communities [29]. Since this is a following process after QIIME analysis, we included the same samples with QIIME (*n* = 1274; Fig. 1). The BIOM format of data from QIIME 1.9 was processed with the PICRUSt version 1.0.0 using the Kyoto Encyclopedia of Genes and Genomes (KEGG) analysis module. Total 328 predicted KOs (KEGG orthology terms) were grouped into the levels of categorization, hierarchical levels 1, 2 and 3. The results were further analyzed with the STAMP version 2.1.3 as a graphical tool [30], extended error bar plot for two-group analysis module of normal/obese groups or box plots for multiple group analysis module of normal/overweight/obese groups. Welch’s t-test for two groups and Kruskal-Walis H-test for multiple groups without controlling of confounding factors were applied. An adjusted *P* value of <0.05 was considered statistically significant after Bonferroni multiple test correction for all analyses.

### Statistical analysis of microbiome data

The zero-inflated Gaussian mixture (fitZIG) model of metagenomeSeq package version 1.14.2 was used for correlation analysis between bacterial normalized count data (as dependent variables) and BMI (as independent categorical variables) [28]. Besides age and sex covariates, dietary components with the strongest impact (Additional file 1: Table S1), and total energy intake were chosen for adjustment according to the residual nutrient model for regression analysis [25, 31]. Since this analysis needs conditions with or without nutrient adjustment, final sample size was 940 after exclusion of missing data on FFQ (Fig. 1). Bacterial count data from QIIME were aggregated to genus level. The genera that were abundant (>50 normalized counts per sample) and prevalent (present in 10% of samples) were applied to the fitZIG model with Bonferroni multiple correction (an adjusted *P* value <0.05 is significant). This analysis was performed using R software package version 3.2.3.

## Results

### Gut bacterial diversity differentiated by BMI category

Table 1 shows descriptive statistics by BMI category. As Fig. 1 presents, final 1274 subjects were included for a basic metagenomic analysis. The relative abundance of gut taxa in each BMI group (normal, overweight, and obese) is considerably even throughout the phylum-to-order level (Additional file 2: Figure S1). The phylum Firmicutes:Bacteroidetes ratio has no significant difference between BMI groups. At family and genus levels, however, bacterial compositional change is seen while processing from normal weight to obese status.

**Fig. 2:**
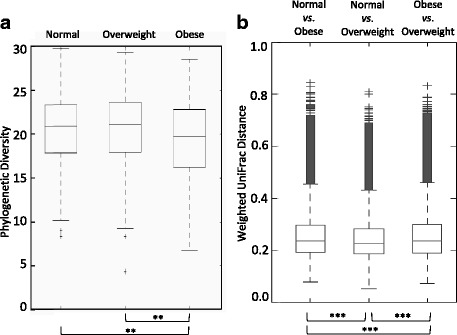
Comparison of (**a**) phylogenetic diversity (PD) across BMI categories, and (**b**) weighted UniFrac distant metrics of each BMI category (^**^ *P* < 0.01, ^***^ *P* < 0.001)

Alpha diversity in OTU level was compared to check the significant difference in diversity. The results shown in Fig. 2a indicate that obese samples have significantly less phylogenetic diversity than normal weight and overweight ones (*P* < 0.01). The overall diversity decreased with increasing BMI.

Distance matrix analysis from Principal Coordinates analysis (PCoA) of weighted UniFrac also identified significant differences between three BMI groups (Fig. 2b) and the statistical significance of sample clustering (ANOSIM; *R* = 0.020, *P* = 0.001).

These results suggest that the diversity according to BMI descends stepwise and the cluster of each BMI group contains unique bacterial components. The distance from normal group was significant greater in obese than overweight group.

### Functional differences of gut microbiota in BMI groups

**Fig. 3:**
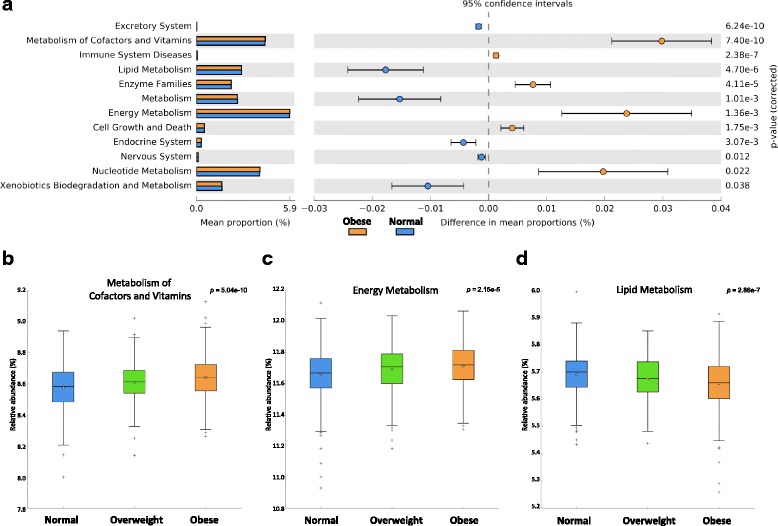
Comparison of PICRUSt predicted KEGG function data based on BMI categories. **a** An extended error bar plot for the comparison of normal vs obese groups. Only functions with *P* < 0.05 are shown. **b**–**d** *Box plots* for multiple group analysis of normal/overweight/obese groups. **b** ‘Metabolism of Cofactors and Vitamins’ (*P* value, 5.04 × 10^−13^), **c** ‘Energy Metabolism’ (2.15 × 10^−5^), **d** ‘Lipid Metabolism’ (2.86 × 10^−7^). *P* value was calculated by Bonferroni multiple test correction methods

PICRUSt analysis identified that ‘Energy Metabolism’ and ‘Metabolism of Cofactors and Vitamins’ genes were over-represented according to BMI increase, as the comparison of obese vs. normal groups (Fig. 3a) as well as in the multi-group comparison with the normal, overweight, and obese groups (Fig. 3b–c) showed a statistical significance. Lipid metabolism, together with excretory and endocrine systems and xenobiotics biodegradation function were depleted in the obese group (Fig. 3a, d). Notably, gene ontologies of essential metabolic pathways in the Metabolism category were present with a reasonable majority (Table 2, Additional file 3: Table S2). We detected predicted increases in genes related to oxidative phosphorylation and purine metabolisms in obese compared to normal-weight subjects. In contrast, we detected decreases in carbohydrate metabolism of glycolysis/gluconeogenesis, pyruvate metabolism, and amino acid metabolism of histidine/arginine-proline/valine-leucine-isoleucine in the obese group. NOD-like receptor signaling, antigen processing and presentation, and primary immunodeficiency involved in inflammation and immune response had significantly higher predicted abundances in the obese group compared with the normal group (Table 2). This result was also true for the three-group comparison of the normal, overweight, and obese groups, in which the immune-related pathways get over-represented with ascending BMI level (Additional file 3: Table S2).

**Table 2:** PICRUSt predicted functions of KEGG categories represented in obese group compared to normal group

KO Functional Categories		Difference between means (95% CI)^a^ obese vs. normal	Adj. *P* values^b^
Level 2	Level 3		
Metabolism of Cofactors and Vitamins	Porphyrin and chlorophyll metabolism	0.025 (0.014–0.037)	7.42 × 10^−3^
Nucleotide Metabolism	Purine metabolism	0.026 (0.014–0.037)	2.36 × 10^−3^
Energy Metabolism	Oxidative phosphorylation	0.020 (0.014–0.027)	1.33 × 10^−7^
	Photosynthesis proteins	0.019 (0.012–0.025)	3.73 × 10^−6^
	Photosynthesis	0.019 (0.012–0.025)	5.64 × 10^−6^
Enzyme Families	Peptidases	0.020 (0.011–0.029)	3.29 × 10^−3^
Amino Acid Metabolism	Histidine metabolism	−0.015 (−0.020–0.009)	1.39 × 10^−4^
	Arginine and proline metabolism	−0.015 (−0.022–0.008)	9.83 × 10^−3^
	Valine, leucine and isoleucine biosynthesis	−0.015 (−0.022–0.008)	2.72 × 10^−5^
Carbohydrate Metabolism	Glycolysis / Gluconeogenesis	−0.013 (−0.019–0.008)	6.39 × 10^−6^
	Pyruvate metabolism	−0.022 (−0.030–0.015)	1.23 × 10^−6^
Immune System Diseases	Primary Immunodeficiency	0.204 (0.139–0.269)	3.21 × 10^−7^
Immune System	Antigen Processing and Presentation	0.068 (0.044–0.092)	7.26 × 10^−6^
	NOD-like receptor signaling pathway	0.064 (0.049–0.080)	8.71 × 10^−13^

Only significant (Adj. *P* < 0.05) level 3 functions for obese vs normal weight were included in this table

^a^Compared the difference between the means of relative frequency (%) of functional trait in normal and obese groups

^b^Applied by Bonferroni multiple comparison correction methods

### Taxonomic comparison by BMI

A genus-level representation of the three BMI categories was assessed by metagenomeSeq, with sequence count data as a dependent variable and BMI as a categorical independent variable, and with controlling of confounding factors.

Statistical analysis using sequence-counting data is challenged by the assumption of normal distribution. This challenge becomes a critical issue when the dependent variable is over-dispersed and contains many instances of zero microbiome count data. We therefore used the zero-inflated Gaussian mixture model in a metagenomeSeq package, which is a recently described and relevant statistical model that is purported to be able to overcome this limitation [28].

Table 3 shows significant differential bacterial genus out of total 87 genera with adjustment for age and sex, with or without nutrient adjustment. Total calorie intake and the one nutrient factor that associated the most were chosen for nutrient adjustment (Additional file 1: Table S1). Strong positive associations of Cyanobacteria and *Desulfovibrio* in overweight subjects compared with normal subjects disappeared after adjustment for intake of fat and fiber, respectively. Cyanobacteria, a hydrogen producer, and *Desulfovibrio*, a sulfate-reducing hydrogentroph, are known to relate with host energy metabolism [11, 13]. Paraprevotellaceae CF231 and Bacteroidales unknown family and unknown genus belong to the order Bacteroidales under the phylum Bacteroidetes, and both had a commonly positive correlation the overweight group. In particular, this positive correlation remained high for Paraprevotellaceae (Adj. *P* value <0.0001) with or without adjustment of fat intake. In contrast, this positive association in the overweight group was not observed in the obese group with an additional adjustment of fat intake. *Acidaminococcus* was the only bacteria that was associated in common throughout all combinations of comparison. Although its positive association with the overweight group disappeared with adjustment of fiber intake, it showed a highly significant positive correlation (Adj. *P* value <0.0001) in the obese group compared with overweight and normal groups. *Eggerthella* negatively associated with both overweight and obese groups compared with the normal group, but the negative association no longer reached statistical significance after adjustment of carbohydrate. The effect estimates were made more significant by adjustment for nutrient intake when the obese group was compared with the overweight group; for example, in the cases of *Acidaminococcus* and *Mitsuokella*. In some cases, like *Akkermansia* and *Adlercreutzia*, there was a nutrient-independent association (Additional file 1: Table S1). The decrease of *Akkermansia*, depletion of which is responsible for causing inappropriate immune responses in the host [32], was significantly associated with obese group compared with the overweight group. Christensenellaceae, which is reported to be associated with leanness [33], showed negative correlation with the obese group only without nutrient adjustment. Additionally, T2DM status or medication of T2DM almost didn’t influence to all these correlations showing in Table 3 (Additional files 4 and 5: Tables S3 and S4).

**Table 3:** Regression analysis between gut microbiota and BMI levels

Overweight vs. Normal	Age- and sex- adjusted		Multivariate adjusted		
	Coefficient^a^	Adj. *P* value^b^	Coefficient^a^	*P* value	Adj. *P* value^b^
Cyanobacteria YS2^c^	0.618	2.63 × 10^−9^	0.035	0.700	1
*Desulfovibrio* ^d^	0.435	9.08 × 10^−5^	−0.101	0.273	1
Bacteroidales unknown family unknown genus^d^	0.403	2.48 × 10^−4^	0.314	0.001	0.068
Paraprevotellaceae CF231^c^	0.360	4.58 × 10^−4^	*0.463*	**2.13 × 10** ^**−7**^	**1.51 × 10** ^**−5**^
*Acidaminococcus* ^d^	0.331	0.002	−0.073	0.403	1
Lactobacillales unknown family unknown genus^c^	0.174	0.031	0.080	0.333	1
*Lactococcus* ^e^	0.152	0.042	0.099	0.064	1
*Eggerthella* ^e^	−0.155	0.005	−0.103	0.022	1
Obese vs. Normal					
*Acidaminococcus* ^d^	0.498	3.90 × 10^−8^	*0.378*	**2.89 × 10** ^**−5**^	**0.002**
Paraprevotellaceae CF231^c^	0.463	7.32 × 10^−6^	0.284	0.003	0.181
*Megasphaera* ^e^	0.443	1.79 × 10^−5^	0.355	0.002	0.146
*Mitsuokella* ^c^	0.302	0.034	0.217	0.013	0.946
*Eggerthella* ^e^	−0.162	0.003	−0.073	0.356	1
Christensenellaceae unknown genus^d^	−0.152	0.031	−0.055	0.003	0.230
Clostridiales unknown family unknown genus	−0.063	0.004	*−0.063*	**5.24 × 10** ^**−5**^	**0.004**
Obese vs. Overweight					
*Acidaminococcus* ^d^	0.329	0.001	*0.504*	**2.64 × 10** ^**−8**^	**1.87 × 10** ^**−6**^
*Mitsuokella* ^c^	0.271	0.007	*0.381*	**1.10 × 10** ^**−6**^	**2.61 × 10** ^**−5**^
*Akkermansia*	−0.225	0.038	*−0.225*	**0.001**	**0.038**
Christensenellaceae unknown genus^d^	−0.179	0.003	−0.170	0.020	0.126
*Adlercreutzia*	0.139	0.007	*0.139*	**9.34 × 10** ^**−5**^	**0.007**

^a^Coefficient (log2 ratio) driven by zero-inflated Gaussian mixture model (fitZig) using metageomeSeq package

^b^Applied by Bonferroni multiple comparison correction

^c^Additionally adjusted for fat and total calorie intake

^d^Additionally adjusted for fiber and total calorie intake

^e^Additionally adjusted for carbohydrate and total calorie intake

Coefficient with Adj. *P* value < 0.05 shown in italic

Remarkably, in comparisons of the obese vs. overweight groups, nutrient adjustment had little effect on the significance; i.e. bacterial components related to the obese group were not influenced by the diet confounding factor compared with overweight group. This suggests there is a signature bacterium for the obesity that has no relation with dietary intake.

## Discussion

Recent human microbiome project studies have linked human gut microbes to obesity, proving the evidence that gut microbiota plays an important role in the harvesting, storage, and expenditure of energy obtained from diet [4, 34]. Our cross-sectional study aimed to identify differences in human gut microbiota associated with BMI in a large-scale metagenome cohort controlled by diet intake information.

Our results, like those of many others, do not support the hypothesis that an increased ratio of Firmicutes to Bacteroidetes may make a significant contribution to the pathophysiology of obesity. However, there is a consistent alpha diversity trend in previous reports that obese individuals have less diverse gut microbiota than normal weight individuals [4]. Clustering of three groups showed a significant difference between each other, with the obese group showing the greatest differences from normal and overweight groups.

The theory of increased energy harvesting by an obesogenic microbiome is supported by the finding of increased production of SCFAs in the obese subjects [10, 13]. Our PICRUSt results indicate that gut microbial function in the obese group involves oxidative phosphorylation which can stimulate lipogenesis or gluconeogenesis [35] while decreasing carbohydrate metabolism. SCFA can increase oxidative phosphorylation, glycolysis, and fatty acid synthesis, which contribute the energy production [36]. SCFAs are generated by microbial fermentation of indigestible dietary polysaccharides into absorbable monosaccharides, which are converted to more complex lipids in the liver [8]. The major SCFAs are acetate, propionate, and butyrate, and the rate and amount of their production depends on the species and amounts of microbes present in colon [37]. Firmicutes, including Clostridium and Lactobacilli, are major producers of acetate and butyrate. Whereas Bacteroidetes can ferment carbohydrate to produce propionate, *Acidaminococcus*, *Megasphaera*, and *Mitsuokella* from the Veillonellaceae family cannot digest a carbohydrate, but can utilize lactate to produce propionate [38]. Our results showed that carbohydrate metabolism in the KEGG pathway was less predicted in the obese group compared with the normal group, which can be speculated by the positive association of Veillonellaceae in the obese group. Paraprevotellaceae (Bacteroidetes) in the overweight group and Veillonellaceae in the obese group contribute to propionate formation but via different pathways, which suggests that substrates or conditions specific to the obese group influence this switch of propionate producers. The mechanism behind this phenomenon will need to be further studied.

Additional mechanisms involving perturbation of the intestinal microbiota and changes in intestinal permeability as potential triggers of inflammation contribute to the risk of obesity and associated diseases [5]. A reduced abundance of *Akkermansia* may reflect a thin mucus layer and thus an impaired gut barrier function with increased translocation of pro-inflammatory bacterial toxins that potentially lead to metabolic disturbances [32]. Lately, *Akkermansia* was proposed to increase body thermogenesis and energy expenditure in cold temperatures [39]. One longitudinal study showed that successful weight reduction in obese human individuals is accompanied by increased *Akkermansia* numbers in feces [40]. A significant negative correlation of *Akkermansia* in the obese group was a consistent feature in our results as well. Thus, this microbe would need to be considered in relation with obesity in future studies.

*Eggerthella* and *Adlercreutzia* in the Coriobacteriia group within Actinobacteria have been repeatedly linked to positive effects in host lipid metabolism and involved in the stimulation of a major hepatic detoxification activity [41]. In addition, these strains have been shown to play a role in the transformation from soy compound to equol, which has higher binding affinity to human estrogen receptors and induces transcription more strongly than any other isoflavone [42]. Our results showed the negative correlation of *Eggerthella* with overweight and obese groups compared to the normal group, but the negative correlation was not significant when adjustment was made for carbohydrate intake. In contrast, the increase of *Adlercreutzia* was significantly correlated with the obese group compared with the overweight group and was not influenced by any nutrients. It can be speculated that *Adlercreutzia* may be replaced in the same niche as *Eggerthella*, but the meaning of this exchange in the obese group will need further study.

We have several limitations from 16S amplicon-based sequencing data which can introduce biases through PCR amplification steps, and resolute only genus level as a maximum [43]. Another limitation of our study could be that our functional approach is represented only by using 16S rRNA gene. However, previous report showed this phylogenetic marker gene, 16S rRNA gene, is sufficiently linked with PICRUSt functional data, which accuracy already reached a maximum with around 100 sequence depth of 16S sequencing [29]. Nevertheless, further studies on the correlation between significant bacteria and their predicted function will be required to define the role related with obesity.

## Conclusions

Although there are a lot of gut microbiota studies regarding obesity, only recently have there been studies using large-scale epidemiologic data with significant statistical power and long-term diet confounding factors. The results of this study will contribute to establishment of a consistent theory on the extent of the influence of intestinal microbiota on obesity. The expectation is that a huge dataset affords the new possibility to discover a novel microbial component with impact on the human health.

## Additional files

Additional file 1: Table S1. Correlations between BMI-associated bacterial genus and dietary intake. (DOC 45 kb)

Additional file 2: Figure S1. Area chart of proportional abundance from phylum down to genus levels in three BMI categories. (a) phylum, (b) class, (c) order, (d) family, (e) genus. Each chart was sorted continuously by ‘Normal ➔ Obese’, from lowest to highest participants. Each color represents a different taxonomic group in the corresponding level. Taxonomical legends of each color in each corresponding level were followed in next slides. (PPT 510 kb)

Additional file 3: Table S2. PICRUSt predicted list of KEGG hierarchical level 3 categories in parent hierarchical level 1 by multiple group comparison. Only significant data of adjusted *P*-value were included. (XLSX 17 kb)

Additional file 4: Table S3. Comparison of regression analysis with inclusion and exclusion of T2DM or T2DM under medication (Med of T2DM). (DOCX 19 kb)

Additional file 5: Table S4. Comparison of regression analysis with or without adjustment of T2DM or T2DM under medication as covariates. (DOCX 19 kb)
