# Supplementary material for: Comparative analysis of gut microbiota associated with body mass index in a large Korean cohort
Source: BMC Microbiol. 2017 Jul 4;17:151. doi: 10.1186/s12866-017-1052-0 (PMC5497371; doi:10.1186/s12866-017-1052-0)
Supplement: Supplementary file 1 — Correlations between BMI-associated bacterial genus and dietary intake. (DOC 45 kb) [file 12866_2017_1052_MOESM1_ESM.doc]

| Phylum | Family | Genus | CHO | FIB | PRO | FAT | Total Calorie |
| --- | --- | --- | --- | --- | --- | --- | --- |
| Actinobacteria | Coriobacteriaceae | Adlercreutzia | 0.049 (0.197) | -0.027 (0.536) | 0.035 (0.320) | -0.022 (0.594) | -0.005 (0.903) |
|  |  | Eggerthella | **0.143 (5.60E-04)** | 0.056 (0.214) | -0.011 (0.804) | -0.048 (0.261) | 0.086 (0.046) |
| Bacteroidetes | Bacteroidales unknown family | Unknown genus | -0.132 (0.109) | **-0.490 (3.53E-07)** | 0.154 (0.057) | -0.012 (0.836) | -0.055 (0.508) |
|  | Paraprevotellaceae | CF231 | -0.060 (0.447) | 0.104 (0.228) | 0.034 (0.642) | **0.519 (4.49E-09)** | 0.420 (4.69E-08) |
| Cyanobacteria | 4C0d-2/YS2/unknown family | Unknown genus | 0.056 (0.449) | -0.152 (0.095) | -0.148 (0.052) | **0.316 (8.88E-05)** | -0.505 (1.28E-09) |
| Firmicutes | Clostridiales unknown family | Unknown genus | 0.018 (0.282) | 0.023 (0.228) | -0.001 (0.937) | 0.004 (0.836) | 0.0002 (0.987) |
|  | Christensenellaceae | Unknown genus | 0.022 (0.746) | **-0.170 (6.58E-04)** | -0.116 (0.019) | -0.009 (0.895) | -0.071 (0.268) |
|  | Lactobacillales unknown family | Unknown genus | 0.269 (6.36E-04) | 0.171 (0.001) | 0.145 (0.004) | **-0.083 (8.88E-05)** | -0.099 (0.198) |
|  | Streptococcaceae | Lactococcus | **0.168 (0.002)** | -0.127 (0.121) | -0.009 (0.858) | -0.039 (0.482) | -0.162 (0.003) |
|  | Veillonellaceae | Acidaminococcus | -0.328 (2.69E-05) | **-0.490 (1.02E-08)** | -0.162 (0.043) | 0.180 (0.019) | 0.004 (0.959) |
|  |  | Megasphaera | **-0.349 (0.001)** | -0.030 (0.673) | -0.061 (0.402) | 0.113 (0.222) | -0.419 (4.66E-06) |
|  |  | Mitsuokella | -0.129 (0.106) | 0.041 (0.650) | 0.282 (3.44E-04) | **0.380 (3.58E-06)** | -0.320 (7.00E-05) |
| (Delta)Proteobacteria | Desulfovibrionaceae | Desulfovibrio | -0.176 (0.020) | **-0.390 (3.84E-06)** | -0.018 (0.813) | 0.374 (5.30E-06) | 0.330 (8.73E-05) |
| Verrucomicrobia | Verrucomicrobiaceae | Akkermansia | 0.148 (0.282) | 0.115 (0.125) | 0.091 (0.179) | -0.035 (0.624) | -0.152 (0.027) |

Additional file 1: Table S1. Correlations between BMI-associated bacterial genus and dietary intake. Log2 ratio coefficient calculated by zero-inflated Gaussian mixture model using metageomeSeq package was shown (*P* value).

The bold letter indicates the strongest significant component.

CHO; carbohydrate, FIB; fiber, PRO; protein, FAT;fat
