# Supplementary material for: Comparative analysis of gut microbiota associated with body mass index in a large Korean cohort
Source: BMC Microbiol. 2017 Jul 4;17:151. doi: 10.1186/s12866-017-1052-0 (PMC5497371; doi:10.1186/s12866-017-1052-0)
Supplement: Supplementary file 2 — Area chart of proportional abundance from phylum down to genus levels in three BMI categories. (a) phylum, (b) class, (c) order, (d) family, (e) genus. Each chart was sorted continuously by ‘Normal ➔ Obese’, from lowest to highest participants. Each color represents a different taxonomic group in the corresponding level. Taxonomical legends of each color in each corresponding level were followed in next slides. (PPT 510 kb) [file 12866_2017_1052_MOESM2_ESM.ppt]

## Slide 1
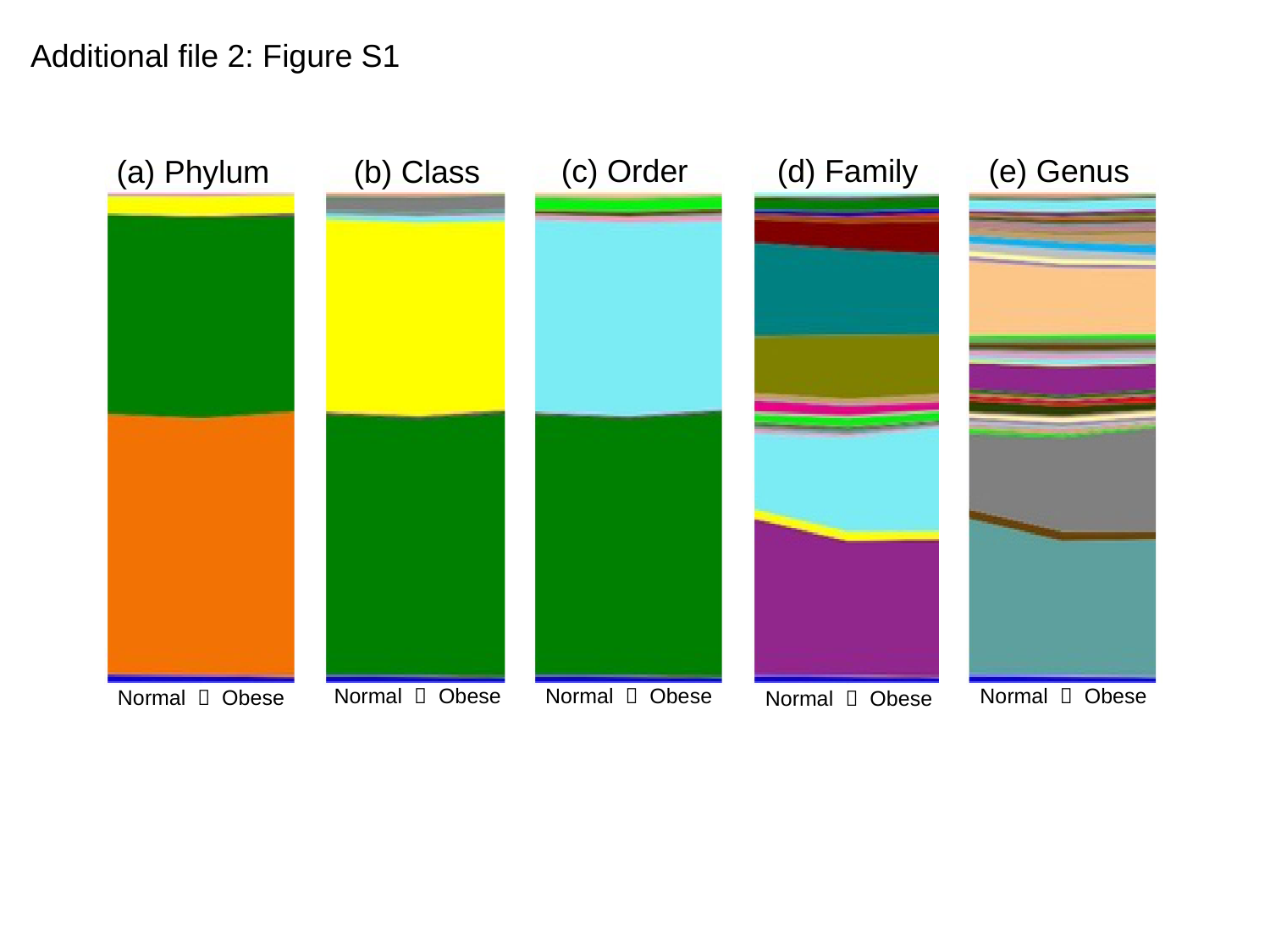

Additional file 2: Figure S1
(c) Order
(d) Family
(e) Genus
(a) Phylum
(b) Class
Normal  Obese
Normal  Obese
Normal  Obese
Normal  Obese
Normal  Obese

## Slide 2
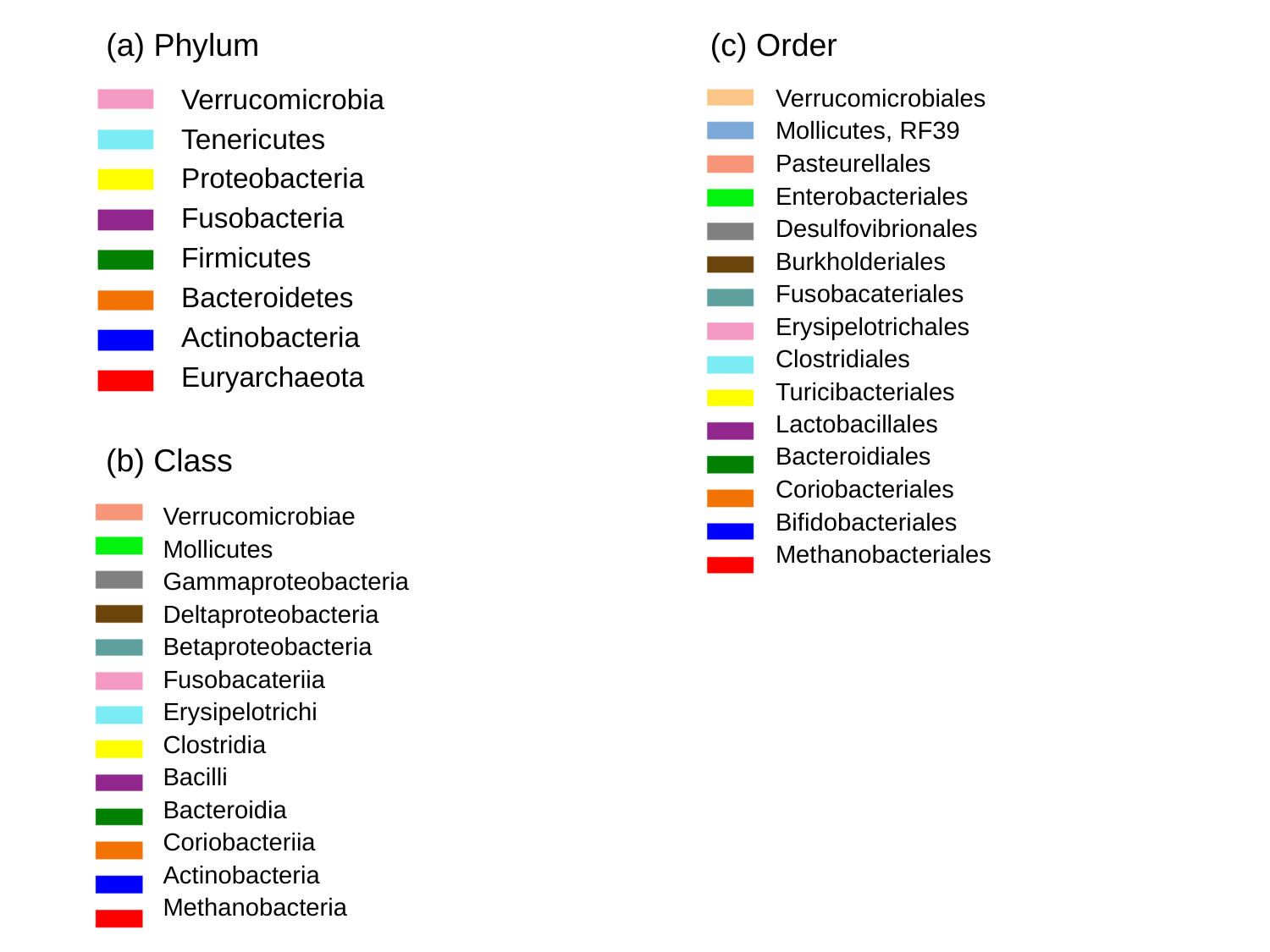

(a) Phylum
(c) Order
Verrucomicrobia
Tenericutes
Proteobacteria
Fusobacteria
Firmicutes
Bacteroidetes
Actinobacteria
Euryarchaeota
Verrucomicrobiales
Mollicutes, RF39
Pasteurellales
Enterobacteriales
Desulfovibrionales
Burkholderiales
Fusobacateriales
Erysipelotrichales
Clostridiales
Turicibacteriales
Lactobacillales
Bacteroidiales
Coriobacteriales
Bifidobacteriales
Methanobacteriales
(b) Class
Verrucomicrobiae
Mollicutes
Gammaproteobacteria
Deltaproteobacteria
Betaproteobacteria
Fusobacateriia
Erysipelotrichi
Clostridia
Bacilli
Bacteroidia
Coriobacteriia
Actinobacteria
Methanobacteria

## Slide 3
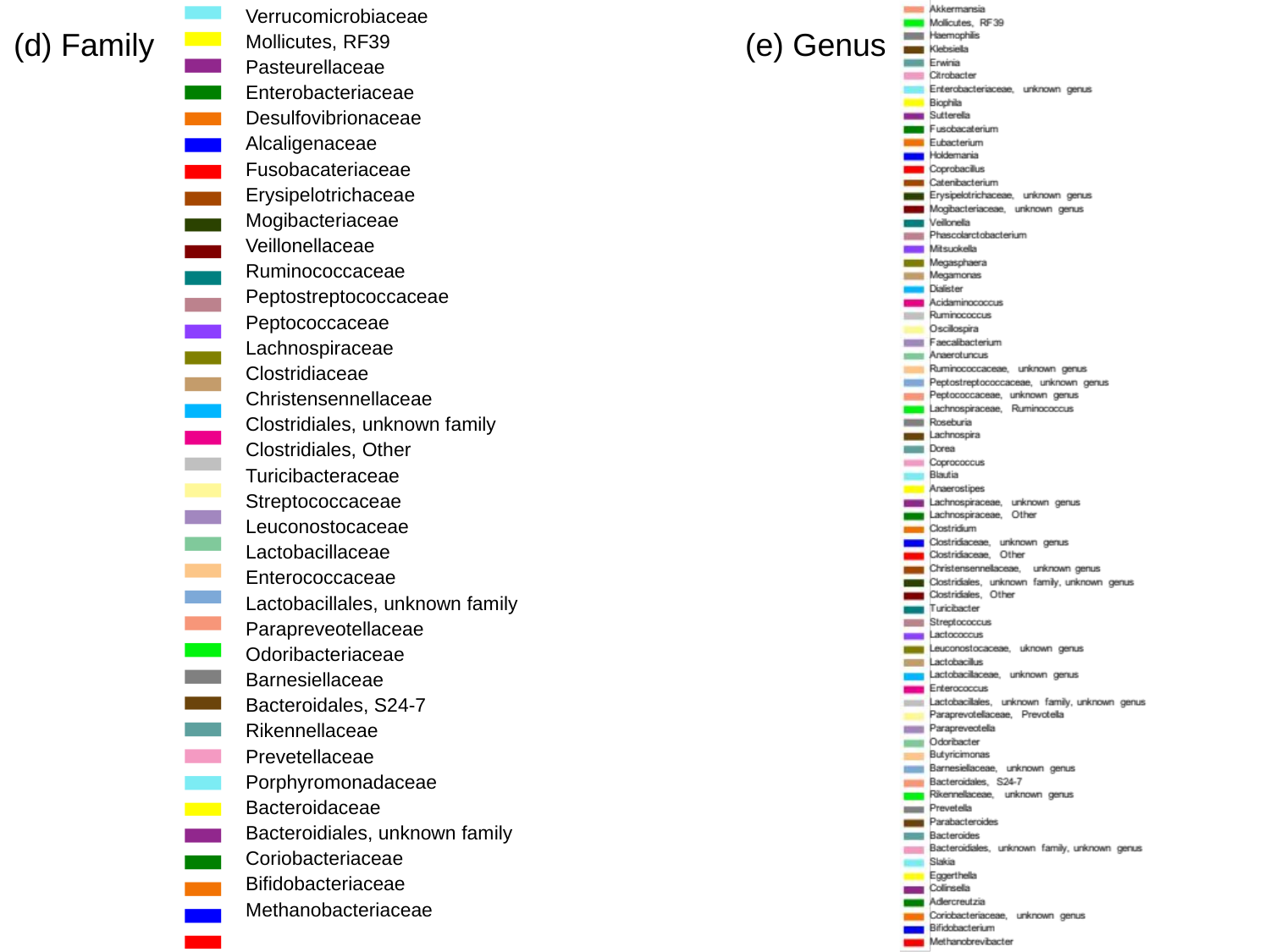

Verrucomicrobiaceae
Mollicutes, RF39
Pasteurellaceae
Enterobacteriaceae
Desulfovibrionaceae
Alcaligenaceae
Fusobacateriaceae
Erysipelotrichaceae
Mogibacteriaceae
Veillonellaceae
Ruminococcaceae
Peptostreptococcaceae
Peptococcaceae
Lachnospiraceae
Clostridiaceae
Christensennellaceae
Clostridiales, unknown family
Clostridiales, Other
Turicibacteraceae
Streptococcaceae
Leuconostocaceae
Lactobacillaceae
Enterococcaceae
Lactobacillales, unknown family
Parapreveotellaceae
Odoribacteriaceae
Barnesiellaceae
Bacteroidales, S24-7
Rikennellaceae
Prevetellaceae
Porphyromonadaceae
Bacteroidaceae
Bacteroidiales, unknown family
Coriobacteriaceae
Bifidobacteriaceae
Methanobacteriaceae
(d) Family
(e) Genus
