# Supplementary material for: Comparative analysis of gut microbiota associated with body mass index in a large Korean cohort
Source: BMC Microbiol. 2017 Jul 4;17:151. doi: 10.1186/s12866-017-1052-0 (PMC5497371; doi:10.1186/s12866-017-1052-0)
Supplement: Supplementary file 4 — Comparison of regression analysis with inclusion and exclusion of T2DM or T2DM under medication (Med of T2DM). (DOCX 19 kb) [file 12866_2017_1052_MOESM4_ESM.docx]

[Additional file 4: Table S3]

^a^ Adjusted for age and sex, plus diet when it’s applicable ^d,e,f^.

| **Overweight *vs.* Normal** | Original result ^a^ | |  | T2DM excluded ^a^ | |  | Med of T2DM excluded ^a^ | | | |
| --- | --- | --- | --- | --- | --- | --- | --- | --- | --- | --- |
|  | Coefficient^b^ | Adj. *P* value^c^ |  | Coefficient^b^ | Adj. *P* value^c^ |  | | Coefficient^b^ | | Adj. *P* value^c^ |
| Cyanobacteria YS2^d^ | 0.035 | 1 |  | -0.029 | 1 |  | 0.012 | | 1 | |
| Desulfovibrio^e^ | -0.101 | 1 |  | -0.145 | 1 |  | -0.098 | | 1 | |
| Bacteroidales unknown family unknown genus^e^ | 0.314 | 0.068 |  | 0.299 | 0.147 |  | 0.302 | | 0.110 | |
| Paraprevotellaceae CF231^d^ | **0.463** | **1.51×10^-5^** |  | **0.496** | **7.76×10^-6^** |  | **0.520** | | **2.47×10^-6^** | |
| Acidaminococcus^e^ | -0.073 | 1 |  | -0.008 | 1 |  | -0.036 | | 1 | |
| Lactobacillales unknown family unknown genus^d^ | 0.080 | 1 |  | 0.065 | 1 |  | 0.079 | | 1 | |
| Lactococcus^f^ | 0.099 | 1 |  | 0.120 | 1 |  | 0.126 | | 1 | |
| Eggerthella^f^ | -0.103 | 1 |  | -0.066 | 1 |  | -0.077 | | 1 | |
| **Obese *vs.* Normal** |  |  |  |  |  |  |  | |  | |
| Acidaminococcus^e^ | **0.378** | **0.002** |  | **0.368** | **0.005** |  | **0.373** | | **0.004** | |
| Paraprevotellaceae CF231^d^ | 0.284 | 0.181 |  | **0.364** | **0.014** |  | **0.340** | | **0.032** | |
| Megasphaera^f^ | 0.355 | 0.146 |  | 0.352 | 0.175 |  | 0.383 | | 0.069 | |
| Mitsuokella^d^ | 0.217 | 0.946 |  | 0.238 | 1 |  | 0.221 | | 1 | |
| Eggerthella^f^ | -0.073 | 1 |  | -0.022 | 0.782 |  | -0.025 | | 1 | |
| Christensenellaceae unknown genus^e^ | -0.055 | 0.230 |  | -0.078 | 1 |  | -0.089 | | 1 | |
| Clostridiales unknown family unknown genus | **-0.063** | **0.004** |  | -0.050 | 0.546 |  | -0.052 | | 0.061 | |
| **Obese *vs.* Overweight** |  |  |  |  |  |  |  | |  | |
| Acidaminococcus^e^ | **0.504** | **1.87×10^-6^** |  | **0.413** | **0.0005** |  | **0.465** | | **2.47×10^-5^** | |
| Mitsuokella^d^ | **0.381** | **2.61×10^-5^** |  | **0.381** | **1.83×10^-4^** |  | **0.387** | | **0.001** | |
| Akkermansia | **-0.225** | **0.038** |  | **-0.258** | **0.011** |  | **-0.239** | | **0.025** | |
| Christensenellaceae unknown genus^e^ | -0.170 | 0.126 |  | -0.028 | 1 |  | -0.031 | | 1 | |
| Adlercreutzia | **0.139** | **0.007** |  | **0.134** | **0.017** |  | **0.127** | | **0.026** | |

^b^ Coefficient (log2 ratio) driven by zero-inflated Gaussian mixture model (fitZig) using metageomeSeq package.

^c^ Applied by Bonferroni multiple comparison correction.

^d^ Additionally adjusted for fat and total calorie intake.

^e^ Additionally adjusted for fiber and total calorie intake.

^f^ Additionally adjusted for carbohydrate and total calorie intake.
