# Supplementary material for: Comparative analysis of gut microbiota associated with body mass index in a large Korean cohort
Source: BMC Microbiol. 2017 Jul 4;17:151. doi: 10.1186/s12866-017-1052-0 (PMC5497371; doi:10.1186/s12866-017-1052-0)
Supplement: Supplementary file 5 — Comparison of regression analysis with or without adjustment of T2DM or T2DM under medication as covariates. (DOCX 19 kb) [file 12866_2017_1052_MOESM5_ESM.docx]

[Additional file 5: Table S4]

| **Overweight *vs.* Normal** | Original result ^a^ | |  | + T2DM covar. ^a^ | |  | + Med of T2DM covar. ^a^ | | | |
| --- | --- | --- | --- | --- | --- | --- | --- | --- | --- | --- |
|  | Coefficient^b^ | Adj. *P* value^c^ |  | Coefficient^b^ | Adj. *P* value^c^ |  | | Coefficient^b^ | | Adj. *P* value^c^ |
| Cyanobacteria YS2^d^ | 0.035 | 1 |  | 0.068 | 1 |  | 0.075 | | 1 | |
| Desulfovibrio^e^ | -0.101 | 1 |  | -0.100 | 1 |  | -0.103 | | 1 | |
| Bacteroidales unknown family unknown genus^e^ | 0.314 | 0.068 |  | 0.310 | 0.087 |  | **0.383** | | **0.004** | |
| Paraprevotellaceae CF231^d^ | **0.463** | **1.51×10^-5^** |  | **0.469** | **1.18×10^-5^** |  | **0.456** | | **2.24×10^-5^** | |
| Acidaminococcus^e^ | -0.073 | 1 |  | -0.024 | 1 |  | -0.059 | | 1 | |
| Lactobacillales unknown family unknown genus^d^ | 0.080 | 1 |  | 0.082 | 1 |  | 0.072 | | 1 | |
| Lactococcus^f^ | 0.099 | 1 |  | 0.097 | 1 |  | 0.099 | | 1 | |
| Eggerthella^f^ | -0.103 | 1 |  | -0.101 | 1 |  | -0.091 | | 1 | |
| **Obese *vs.* Normal** |  |  |  |  |  |  |  | |  | |
| Acidaminococcus^e^ | **0.378** | **0.002** |  | **0.373** | **0.002** |  | **0.335** | | **0.013** | |
| Paraprevotellaceae CF231^d^ | 0.284 | 0.181 |  | 0.226 | 1 |  | 0.238 | | 0.820 | |
| Megasphaera^f^ | 0.355 | 0.146 |  | 0.355 | 0.145 |  | 0.356 | | 0.143 | |
| Mitsuokella^d^ | 0.217 | 0.946 |  | 0.252 | 1 |  | 0.243 | | 1 | |
| Eggerthella^f^ | -0.073 | 1 |  | -0.067 | 1 |  | -0.049 | | 1 | |
| Christensenellaceae unknown genus^e^ | -0.055 | 0.230 |  | -0.077 | 1 |  | -0.171 | | 1 | |
| Clostridiales unknown family unknown genus | **-0.063** | **0.004** |  | **-0.055** | **0.058** |  | **-0.055** | | **0.045** | |
| **Obese *vs.* Overweight** |  |  |  |  |  |  |  | |  | |
| Acidaminococcus^e^ | **0.504** | **1.87×10^-6^** |  | **0.351** | **0.001** |  | **0.297** | | **0.011** | |
| Mitsuokella^d^ | **0.381** | **2.61×10^-5^** |  | **0.391** | **4.87×10^-5^** |  | **0.375** | | **1.27×10^-4^** | |
| Akkermansia | **-0.225** | **0.038** |  | **-0.229** | **0.031** |  | **-0.234** | | **0.023** | |
| Christensenellaceae unknown genus^e^ | -0.170 | 0.126 |  | -0.028 | 1 |  | -0.027 | | 1 | |
| Adlercreutzia | **0.139** | **0.007** |  | **0.126** | **0.029** |  | **0.125** | | **0.023** | |

^a^ Adjusted for age and sex, plus diet when it’s applicable ^d,e,f^.

^b^ Coefficient (log2 ratio) driven by zero-inflated Gaussian mixture model (fitZig) using metageomeSeq package.

^c^ Applied by Bonferroni multiple comparison correction.

^d^ Additionally adjusted for fat and total calorie intake.

^e^ Additionally adjusted for fiber and total calorie intake.

^f^ Additionally adjusted for carbohydrate and total calorie intake.
